# Secondary Acrodermatitis Enteropathica in a Premature Low-Birth-Weight Infant: A Case Report

**DOI:** 10.7759/cureus.113376

**Published:** 2026-07-25

**Authors:** Noria AlFadhel, Reem AlQusaimi, Alsadat Mosbeh, Abeer Albazali

**Affiliations:** 1 Dermatology, Farwaniya Hospital, Kuwait City, KWT; 2 Dermatology/Dermatopathology, Faculty of Medicine, Al-Azhar University, Cairo, EGY

**Keywords:** acrodermatitis enteropathica, pediatric dermatology, periorificial dermatitis, prematurity, zinc deficiency

## Abstract

Acrodermatitis enteropathica (AE) is a rare disorder of zinc deficiency that can be hereditary or acquired. Secondary AE is uncommon in premature infants and may mimic other inflammatory or infectious dermatoses, making early diagnosis challenging.

A four-month-old premature Egyptian female infant, born at 27 weeks' gestation with a birth weight of 880 g, presented with a one-month history of progressive periorificial and acral dermatitis associated with intermittent diarrhea. The lesions failed to respond to multiple courses of antibiotics. Histopathological findings, together with the clinical presentation and low zinc levels, supported the diagnosis of secondary AE. Oral zinc supplementation resulted in marked clinical improvement.

This case highlights the importance of considering secondary AE in premature, low-birth-weight infants presenting with characteristic dermatitis. Early recognition and timely zinc supplementation are essential to prevent complications and achieve favorable clinical outcomes.

## Introduction

Acrodermatitis enteropathica (AE) is a rare disorder of zinc deficiency characterized by the classic triad of periorificial and acral dermatitis, diarrhea, and alopecia. It occurs as either primary (hereditary) AE, an autosomal recessive disorder caused by mutations in the SLC39A4 gene resulting in impaired intestinal zinc absorption, or secondary (acquired) AE, which develops due to inadequate zinc intake, malabsorption, prematurity, low birth weight, or increased metabolic requirements [1].

Hereditary AE has an estimated incidence of approximately one in 500,000 live births, while secondary AE is more commonly encountered in premature and very-low-birth-weight infants because of limited zinc stores and increased nutritional demands. Early recognition is essential, as prompt zinc supplementation results in rapid clinical improvement and prevents significant morbidity [2]. However, differentiating hereditary from acquired AE at initial presentation may be challenging, as both forms share similar clinical manifestations and often require correlation of the clinical history, laboratory findings, therapeutic response, and, when available, genetic testing.

We report the case of a premature, low-birth-weight infant with secondary AE who presented with progressive periorificial and acral dermatitis and intermittent diarrhea. The diagnosis was supported by characteristic clinical and histopathological findings, low serum zinc levels, and a dramatic response to oral zinc supplementation.

## Case presentation

A four-month-old Egyptian female infant, born prematurely at 27 weeks' gestation via cesarean section with a birth weight of 880 g, presented with a one-month history of a progressively worsening skin eruption. She was exclusively breastfed. One month prior to presentation, her mother noticed increasing irritability, followed by the appearance of an ill-defined erythematous, crusted eruption over the face with yellow crusting around the nose. The infant was initially evaluated at a maternity hospital, where she received topical therapy and oral amoxicillin-clavulanate twice daily for seven days for presumed bacterial skin infection. Despite good treatment compliance, there was no clinical improvement.

One week later, the eruption became more extensive, predominantly affecting the face before spreading to the neck, ears, and acral regions of the upper and lower extremities. The patient was subsequently evaluated and prescribed a five-day course of azithromycin; however, the lesions continued to progress with the development of desquamation, prompting referral to the dermatology service for further assessment.

The mother also reported intermittent non-bloody loose stools during the course of the illness. The infant remained active, tolerated exclusive breastfeeding well, and was gaining weight appropriately. There was no history of fever, recent travel, sick contacts, or pet exposure. The patient had not yet received her scheduled two-month immunizations. Family history was negative for autoimmune disorders, inherited diseases, or dermatological conditions.

**Figure 1:**
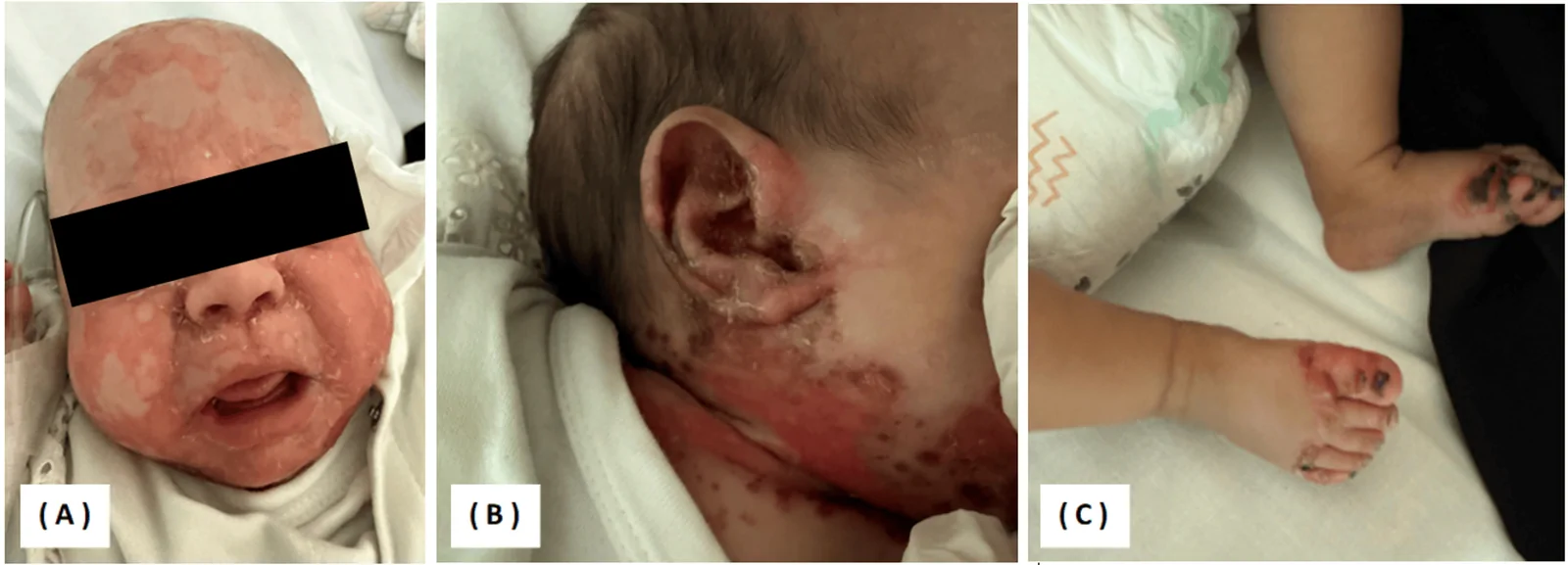
Clinical presentation at the initial evaluation Clinical photographs demonstrating the characteristic cutaneous manifestations at presentation. (A) Diffuse facial erythema with well-demarcated periorificial eczematous plaques, crusting around the nose, and superficial desquamation. (B) Erythematous, crusted, and desquamative plaques involving the left ear, retroauricular region, and lateral neck. (C) Acral involvement with erosive and crusted lesions affecting the bilateral great toes and left thumb.

On examination, the infant appeared irritable and was crying but remained hemodynamically stable. Cutaneous examination revealed well-demarcated erythematous annular eczematous plaques with crusting and superficial desquamation, demonstrating prominent periorificial involvement of the face. Bilateral eyelid erythema with crusting at the medial canthi was noted, in addition to bilateral erythema, crusting, and desquamation involving the ears and retroauricular folds. Diffuse erythema of the neck with areas of crusting and superficial sloughing was also present. Examination of the extremities demonstrated acral involvement with ruptured bullae and crusting affecting both thumbs and great toes (Figure 1). The trunk was spared, and no active genital lesions were identified on examination; however, the mother reported a similar but milder eruption involving the diaper area earlier in the disease course.

Laboratory investigations demonstrated leukocytosis, mild anemia, and reactive thrombocytosis. Renal function tests showed a blood urea level of 1.1 mmol/L, serum creatinine of 19 µmol/L, sodium of 137 mmol/L, and potassium of 6.1 mmol/L. Liver function tests revealed a low alkaline phosphatase level of 101 U/L, while alanine aminotransferase, aspartate aminotransferase, and gamma-glutamyl transferase were within normal limits (Table 1). Serum zinc level measured 7 µmol/L (Table 2). Blood cultures, methicillin-resistant *Staphylococcus aureus *screening, and wound cultures were all negative (Table 3). 

**Table 1:** Hematological and biochemical investigations at presentation Baseline laboratory findings demonstrating leukocytosis, mild anemia, reactive thrombocytosis, and reduced alkaline phosphatase, with otherwise unremarkable renal and hepatic function tests.

Test	Value	Reference range
White blood cell (10^9^/L)	19.3	5-15
Red blood cell (10^12^/L)	4.8	4-5.2
Hemoglobin (g/L)	104	110-130
Platelet count (10^9^/L)	691	200-490
Lymphocytes (10^9^/L)	8	6-9
Creatinine (µmol/L)	19	11-34
Sodium (mmol/L)	137	136-144
Potassium (mmol/L)	6.1	3.6-6.4
Alkaline phosphatase (U/L)	101	150-420
Gamma-glutamyl transferase (U/L)	26	12-122
Alanine aminotransferase (U/L)	16	5-45
Aspartate aminotransferase (U/L)	39	20-80

**Table 2:** Serum zinc level at presentation Serum zinc concentration obtained at initial presentation, supporting the diagnosis of zinc deficiency in conjunction with the clinical, histopathological, and therapeutic findings.

	Value	Reference range
Serum zinc level (µmol/L)	7	10.7-18

**Table 3:** Microbiological investigations at presentation Wound swab culture was negative for potential pathogenic organisms, with no significant bacterial growth detected.

Microbiology	
Specimen	Wound swab culture
Culture yields	No potential pathogen isolated

**Figure 2:**
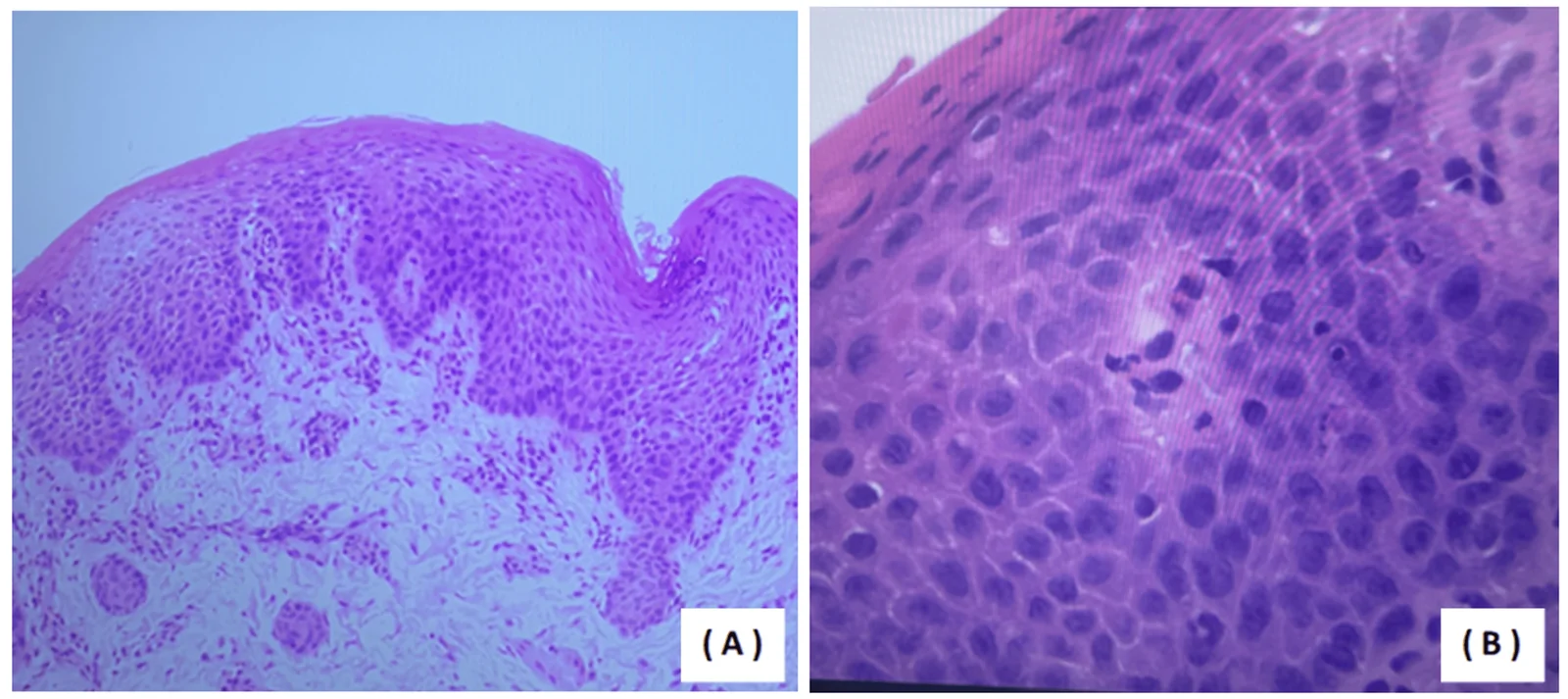
Histopathological findings of acrodermatitis enteropathica Histopathological examination of the skin biopsy. (A) Low-power H&E section demonstrating acanthosis with elongation of the rete ridges, hypogranulosis, and confluent parakeratosis. (B) High-power H&E section showing dyskeratotic keratinocytes within the epidermis. These findings were reported as suggestive of acrodermatitis enteropathica.

A punch biopsy obtained from an active lesion demonstrated psoriasiform epidermal hyperplasia with acanthosis and elongation of the rete ridges, hypogranulosis, parakeratosis, and scattered dyskeratotic keratinocytes within the epidermis. These histopathological findings were consistent with AE (Figure 2). 

**Figure 3:**
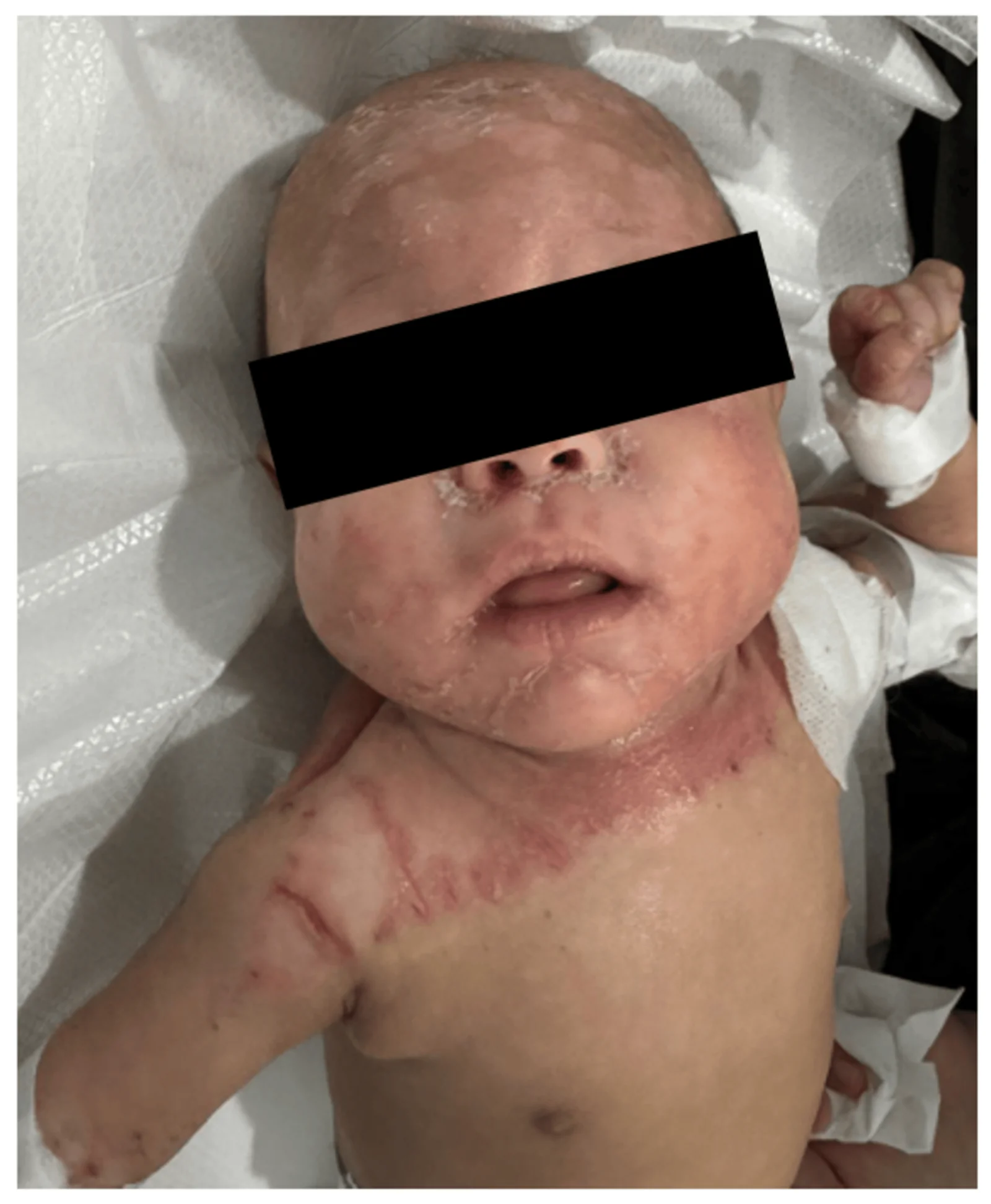
Clinical improvement two weeks after the initiation of oral zinc supplementation Follow-up photograph obtained two weeks after the initiation of oral zinc supplementation, demonstrating marked resolution of the facial and cervical dermatitis with minimal residual scaling.

Based on the characteristic periorificial and acral distribution of the dermatitis, intermittent diarrhea, low serum zinc level, histopathological findings, and the clinical context of prematurity and low birth weight, a diagnosis of secondary AE was considered the most likely diagnosis. Oral zinc sulfate was initiated at a dose of 2 mg/kg/day of elemental zinc. The patient demonstrated marked clinical improvement of the cutaneous lesions (Figure 3) within two weeks of treatment, accompanied by normalization of the serum zinc level to 15 µmol/L after one month of therapy, thereby providing both therapeutic and biochemical confirmation of zinc deficiency.

## Discussion

AE is a rare disorder of zinc deficiency characterized by the classic triad of periorificial and acral dermatitis, diarrhea, and alopecia. It is classified into primary (hereditary) AE, an autosomal recessive disorder caused by mutations in the SLC39A4 gene resulting in impaired intestinal zinc absorption, and secondary (acquired) AE, which occurs due to inadequate zinc intake, malabsorption, prematurity, low birth weight, or increased zinc requirements [3]. Although the complete triad is not always present, patients typically develop well-demarcated erythematous, eczematous, or erosive plaques involving the periorificial, anogenital, and acral regions. Additional manifestations may include irritability, poor wound healing, paronychia, glossitis, and recurrent infections [1]. Our patient demonstrated several of these characteristic features, including progressive periorificial and acral dermatitis, intermittent diarrhea, and irritability in the setting of prematurity and low birth weight, making secondary AE a strong clinical consideration despite its rarity.

Zinc is an essential trace element involved in cellular proliferation, protein synthesis, immune function, wound healing, and epidermal differentiation. In hereditary AE, mutations in the SLC39A4 gene impair the function of the ZIP4 transporter, resulting in defective intestinal zinc absorption and systemic zinc deficiency. In contrast, secondary AE develops when zinc requirements exceed intake or absorption despite an intact transporter. Prematurity and low birth weight are major risk factors because infants are born with limited zinc stores, have increased nutritional requirements during rapid growth, and may have immature intestinal absorption [4]. Additional risk factors include exclusive breastfeeding from mothers with low breast milk zinc concentrations, malabsorption syndromes, prolonged parenteral nutrition, and chronic gastrointestinal disease. These mechanisms culminate in impaired epidermal integrity and the characteristic mucocutaneous manifestations of zinc deficiency [5]. In our patient, several recognized risk factors coexisted, including extreme prematurity (27 weeks' gestation), very low birth weight (880 g), and exclusive breastfeeding, all of which likely contributed to the development of secondary zinc deficiency.

The diagnosis of AE is primarily based on the characteristic clinical presentation, supported by laboratory investigations and response to zinc therapy. Serum zinc concentration is the most commonly used biochemical marker; however, normal serum zinc levels do not exclude the diagnosis because circulating zinc represents only a small fraction of total body zinc and may remain within the reference range despite tissue depletion [6]. Measurement of serum alkaline phosphatase, a zinc-dependent enzyme, can provide additional supportive evidence, while histopathology typically demonstrates psoriasiform epidermal hyperplasia, pallor of the upper epidermis (necrolysis), confluent parakeratosis, spongiosis, and dyskeratotic keratinocytes. In diagnostically challenging cases, a rapid clinical response to oral zinc supplementation is considered a valuable diagnostic tool. In hereditary cases, confirmation can be achieved by identifying pathogenic variants in the SLC39A4 gene [7]. Our patient exhibited the classic periorificial and acral distribution of dermatitis, a reduced serum zinc level (7 µmol/L), low alkaline phosphatase, and histopathological findings consistent with AE. The marked clinical improvement and normalization of serum zinc levels following oral zinc supplementation further supported the diagnosis of zinc deficiency and reinforced the clinical suspicion of secondary AE.

Although the clinical presentation strongly favored secondary AE, several limitations should be acknowledged. Genetic testing for SLC39A4 mutations was not performed; therefore, hereditary AE cannot be definitively excluded. In addition, maternal breast milk zinc concentration was not assessed, and follow-up after the discontinuation of zinc supplementation was unavailable to evaluate for recurrence, which may have further helped distinguish hereditary from acquired disease. Nevertheless, the patient's extreme prematurity, very low birth weight, reduced serum zinc level, characteristic clinicopathological findings, and marked clinical and biochemical response to oral zinc supplementation strongly supported secondary zinc deficiency as the most likely diagnosis.

Oral zinc supplementation is the mainstay of treatment for AE, commonly administered as elemental zinc at approximately 1-3 mg/kg/day, with the dose adjusted according to clinical response and serum zinc levels. Hereditary disease requires lifelong therapy, whereas treatment of acquired disease may be discontinued after the correction of the underlying cause and the restoration of adequate zinc stores. Supportive measures include wound care, nutritional optimization, and treatment of secondary infections. Long-term high-dose zinc therapy may induce copper deficiency; therefore, zinc, copper, complete blood count, and growth parameters should be monitored. Untreated zinc deficiency may lead to growth failure, recurrent infections, impaired wound healing, neurological symptoms, and, in severe cases, sepsis or death. Prognosis is generally excellent with early recognition, as cutaneous lesions frequently improve within days to weeks of supplementation [8]. Our patient demonstrated rapid clinical improvement after oral zinc supplementation, with resolution of the dermatitis and normalization of serum zinc levels, highlighting the excellent prognosis associated with timely recognition and treatment.

## Conclusions

This case highlights the importance of maintaining a high index of suspicion for zinc deficiency presenting as AE, particularly in premature, low-birth-weight infants with persistent periorificial and acral dermatitis. Although the clinical, biochemical, histopathological, and therapeutic findings were most consistent with secondary AE, hereditary disease could not be definitively excluded in the absence of genetic testing and long-term follow-up. Recognition of the characteristic clinical features and associated risk factors is essential to avoid delays in diagnosis and unnecessary therapies. Timely zinc supplementation can result in rapid clinical improvement and favorable outcomes in infants with zinc deficiency.
